# Distinct contextual roles for Notch signalling in skeletal muscle stem cells

**DOI:** 10.1186/1471-213X-14-2

**Published:** 2014-01-24

**Authors:** Philippos Mourikis, Shahragim Tajbakhsh

**Affiliations:** 1 Stem Cells and Development, CNRS URA 2578, Department of Developmental & Stem Cell Biology, Institut Pasteur, 25 rue du Dr. Roux, 75015 Paris, France

**Keywords:** Notch, Skeletal muscle, Quiescence, Regeneration

## Abstract

Notch signalling acts in virtually every tissue during the lifetime of metazoans. Recent studies have pointed to multiple roles for Notch in stem cells during quiescence, proliferation, temporal specification, and maintenance of the niche architecture. Skeletal muscle has served as an excellent paradigm to examine these diverse roles as embryonic, foetal, and adult skeletal muscle stem cells have different molecular signatures and functional properties, reflecting their developmental specification during ontology. Notably, Notch signalling has emerged as a major regulator of all muscle stem cells. This review will provide an overview of Notch signalling during myogenic development and postnatally, and underscore the seemingly opposing contextual activities of Notch that have lead to a reassessment of its role in myogenesis.

## Background

Notch signalling is one the major regulatory pathways that define multicellular development, principally by dictating the fate of one cell in relation to that of its neighbouring cell [1]. This pathway relies on cell-to-cell communication, by virtue of the fact that both the Notch receptors (Notch1-4 in mammals) and their ligands (Delta-like1, Delta-like4, Jagged1, and Jagged2 in mammals) are transmembrane proteins (*note*: Dll3 is not considered, as it does not act as a Notch ligand, [2,3]). The cleaved, intracellular domain of Notch, NICD, is also a transcriptional coactivator [4-6] that interacts with RBPJ [7-10] and induces its binding to DNA to regulate gene expression [11,12].

Although the fundamental role of Notch signalling during development has been well studied, its function in the adult organism was overshadowed until the stem cells of fully-grown and aged organisms became a subject of systematic research. Today, we know that Notch activity is critical for the maintenance of diverse types of adult somatic stem cells, both quiescent and proliferating [13-17]. In the skeletal muscle, adult stem (satellite) cells that are quiescent during homeostasis, have robust regenerative capacity and are regulated by Notch signalling activities.

The origin of satellite cells in the body can be traced back to mesodermal cells of the dermomyotome [18], a transient epithelial structure of the somites formed in the mouse around embryonic day (E)9. These skeletal muscle founder stem cells proliferate and express the paired-box/homeodomain transcription factors Pax3 and Pax7 [19-21]. They undergo myogenic specification following the sequential expression of the bHLH Myogenic Regulatory Factors (MRFs), *Myf5*, *Mrf4, Myod*, and subsequently the differentiation gene *Myogenin *[22-24]. Juvenile satellite cells that are Pax7^+^ are observed under the myofibre basement membrane from E16.5 [20] and they act as reservoirs of adult satellite cells that emerge between 2–3 weeks postnatally [22,25,26].

The main focus of this review is to examine the role of the Notch pathway during the establishment, maintenance, and activation of satellite cells in the mouse. We speculate on the cellular contexts, during mouse development and postnatally, that ensure proper activation of Notch signalling in skeletal muscle stem cells. Moreover, we underscore the opposing contextual outcomes of Notch activity and propose alternative views for its role during quiescence and regeneration.

## Notch receptors and their ligands in myogenic stem cells during development

Notch signalling plays a central role in maintaining muscle stem cells during prenatal and postnatal skeletal muscle development. Therefore, there is a requirement for continuous Notch signalling during the ontogeny of muscle, as well as during adult muscle homeostasis. So, what is the identity and the source of the ligand required to maintain appropriate Notch signalling in the right cells at the right time?

In the embryo, the major player appears to be Delta-like1 (Dll1), provided mainly by the committed myoblasts [13,27-29]. These cells are Myod^+^ that, in analogy to neuronal bHLH factors [30], induces the expression of Dll1 [31] to signal to upstream cells and sustain the stem/progenitor cell population by direct cell-cell interaction. This feedback mechanism of receptor/ligand regulation, utilized in many other cell systems [32,33], is coherent with the transcriptional signature of upstream (high levels of Pax7, “Pax7^Hi^”) and differentiating (Pax7^Low^) myogenic cells that show high levels of Notch receptors (Notch-1, -2,-3) and activity in the former, and high Dll1 ligand expression in the latter [13]. Notably, the Pax7 population in the mouse does not express detectable levels of Dll4, and the transcripts of the Notch ligand and target *Jagged-1* are detectable only in the Pax7^Hi^ cells that receive Notch signalling (PM, ST, unpublished observations).

The prevalence of Dll1 in committed myogenic cells is consistent with the muscle phenotypes in mouse embryos with reduced levels of this ligand (hypomorphic over null *Dll1* allele), where there is severe muscle hypotrophy due to precocious differentiation of the muscle stem cell population [29]. With the caveat that this was a germline mutation and not cell type specific, this study strongly suggests that Dll1 is a necessary and sufficient Notch ligand for the maintenance of myogenic stem cells during embryogenesis. Similarly, loss of myogenic stem cells was accompanied by increased differentiation in mouse embryos upon muscle-specific, conditional deletion of *Rbpj*[34]. In a complementary approach, it was shown that *Myf5*^*Cre*^ driven expression of constitutively active Notch1 (*Myf5*^*Cre*^*:R26*^*stop-NICD*^) maintained upstream myogenic cells undifferentiated and Pax7^+^, even in the absence of differentiating progeny [35]. By extrapolation, we can conclude that Dll1-triggered canonical Notch signalling fulfils the requirements as the principal pathway, sufficient to autonomously maintain embryonic myogenic cells throughout development.

A distinct source of Dll1 ligand was described for a subpopulation of muscle progenitors during early myogenesis in the chick embryo [36]. In that study, migrating neural crest cells expressing Dll1 trigger the transient activation of Notch signalling in muscle progenitors specifically within the dorsal dermomyotome of the somite, as these delaminate to commit to myogenesis. This attractive regulatory mechanism, which involves the coordination of different cell types, remains to be tested in diverse model organisms and in other muscle progenitors outside the dorsomedial lip of the dermomyotome.

## Notch receptors and their ligands in satellite cells

In adult homeostatic muscle, quiescent satellite cells express Notch -1, -2, and -3 as well as high levels of the Notch/Rbpj targets *Hey1*, *HeyL*, and *Hes1*, thereby reflecting high Notch activity. Abrogation of Notch signalling by targeted deletion of *Rbpj* results in the spontaneous differentiation of this cell population. Therefore, satellite cells are sustained in a quiescent state by canonical Notch activity [13,37]. Interestingly, Notch3 germline knock-out mice have a seemingly opposite phenotype, with an abnormally high number of satellite cells and hypertrophic regenerated muscle even after seven rounds of injury, indicating an antagonistic function with the other Notch receptors [38]. Although canonical Notch signalling is transduced by Rbpj, how this transcription factor relays signalling from each of the Notch receptors is a critical question that could unveil further surprises. Conditional deletion of Notch3, as well as that of Notch-1 and -2, would provide useful information for the functional relationship of the Notch paralogues in satellite cell homeostasis.

Based on the anatomical position of adult satellite cells between the myofibre and the basement membrane, the muscle fibre is the most likely source of ligand. However, the lack of reliable mouse Dll1 antibodies has hindered the direct visualization of the protein, especially relative to the position of the satellite cells. Genetic, inducible depletion of Dll1 and/or Dll4 specifically in the myofibres should be performed to validate the main source of the ligand. The basal lamina of the basement membrane, a cell-free extracellular matrix protein rich structure, is located in apposition to the myofibre. Although proteoglycans of the basal lamina bind secreted cytokines and other signalling molecules [39], they are not expected to bind Notch ligands as these are transmembrane proteins and their soluble form is not active [40,41].

Alternatively, several cell types that reside outside the basement membrane, including pericytes, endothelial cells, PICs (Pw1+ interstitial cells), fibro-adipogenic and mesenchymal cells, could potentially act as source of ligand [42]. In addition, satellite cells have been shown to be closely associated to capillaries of human and mouse muscle [43]. Though no apparent physical contact has been demonstrated yet between satellite and endothelial or pericyte cells, the latter cell types might contribute to Notch activation in the satellite cells. Indeed, both in vertebrates and invertebrates Dll-bearing cellular protrusions (filopodia) capable of activating Notch signalling at a long-range have been described [44-46], providing a possible mode of cell interactions crossing the basement membrane. Moreover, even in the absence of cell contact, soluble factors secreted by interstitial cells might enhance stimulation of the Notch pathway in satellite cells by a paracrine mechanism.

The muscle fibre, its ensheathing basement membrane, as well as the various cell types indicated above provide a complex microenvironment that maintains satellite cells in a G_0_ reversible cell cycle state, whilst retaining their extraordinary regenerative potential. Disruption of this satellite cell niche invariably leads to exit from quiescence and entry into a phase of active proliferation. The status of Notch signalling in satellite cells during the transition from G_0_-exit to the proliferation of myogenic progeny cells has been difficult to decipher. Recent studies point to a more complex role than previously anticipated.

## Proliferating and Quiescent myogenic stem cells: two distinct cell states regulated by Notch

In the mouse embryo, Notch signalling is essential for the maintenance of proliferating Pax7^+^ cells, although it appears not to be required for their emergence in the dermomyotome [29,34]. In the adult, active Notch signalling is critical for sustaining quiescent satellite cells during homeostasis [13,37]. In addition to maintaining quiescence, Notch activity is likely to facilitate the transition into that cell state, as forced expression of NICD promotes precocious cell cycle exit prenatally, characterised by the quiescence marker signature: Pax7^+^, CalR^+^ (CalR: Calcitonin receptor, marker of adult satellite cells), Myod^— ^[35]. To date, it is unclear how Notch maintains stem cells in a proliferative state during development and in a quiescent state in the adult. One common feature is by preventing differentiation thereby raising the question whether the quiescent state reflects an absence of proliferation and differentiation or a *bona fide* defined cell state.

## Notch signalling collapses during satellite cell activation: a prerequisite or consequence?

In the context of skeletal muscle repair, previous reports suggested that satellite cell activation and subsequent proliferation is driven by Notch signalling [47-49]. This notion was in agreement with the role of this signalling pathway during development. More recent studies, however, have fundamentally changed this view by examining the activity of the Notch pathway directly in myogenic cells *in vivo*. Here we highlight the recent reports that point to a more nuanced role for Notch during adult myogenesis. We will first describe the key steps that characterise satellite cell activation, then discuss the role of Notch signalling during these critical stages.

One feature of G_0_-exit is the prolonged G1 phase that follows the breaking of quiescence. This is indeed the case for satellite cells: in injured muscles, the first S-phase of activated satellite cells *in vivo* initiates between 14 h and 18 h post-injury (Notexin-injury), [50]. Accordingly, the first mitoses of quiescent satellite cells isolated by FACS are observed between 18-27 h after plating in low oxygen [13,50]. To date, the earliest molecular marker of activated satellite cells is phosphorylated p38 (pp38α/β), followed by Myod [51-53]. Also, *Myf5* is highly upregulated upon activation [54,55], but unlike Myod, Myf5 protein is detectable in the majority of quiescent satellite cells [55]. Interestingly, during the G1-phase following quiescence, Myod does not promote differentiation, but instead it directly regulates the expression of *Cdc6*, a gene involved in rendering chromatin accessible for DNA replication [53,56]. Once activated, myoblasts enter the cell cycle and continue to divide every 7-8 h (both *in vivo* and in culture in low O_2_; [50]) until the majority progress to terminal differentiation by downregulating *Pax7* and upregulating *Myogenin*. A fraction of the proliferating population, however, is able to self-renew and return to quiescence (Pax7^+^/Myod^-^).

It was previously proposed that Notch signalling is the driving force for the proliferation of activated satellite cells, and this notion was consistent with the general pro-mitotic action of NICD in other systems [57,58]. However, recent experimental evidence has revised our view of the role of Notch signalling during activation. These are based on: 1) the drastic decrease of endogenous Notch activity *in vivo* following muscle injury; 2) the continued proliferation and efficient regeneration of *Rbpj* null satellite cells; 3) the hindered proliferation of activated satellite cells that express constitutively active NICD.

**Figure 1:**
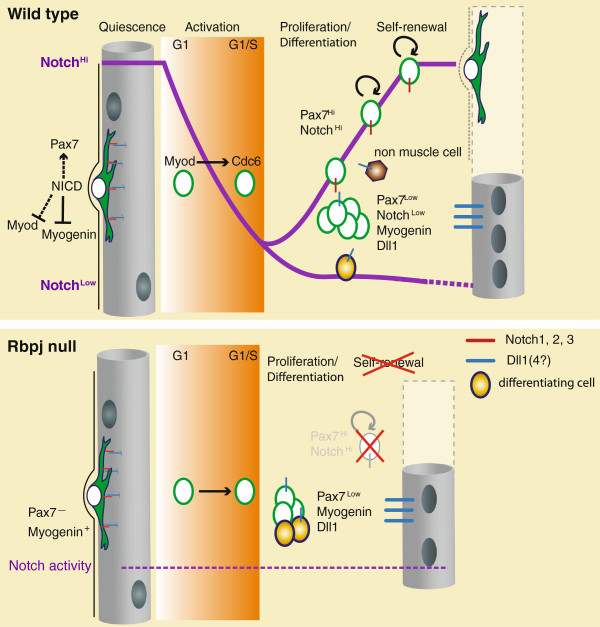
**Notch signalling activity during muscle regeneration. ***Upper panel:* In wild type muscle, quiescent, G_0_-arrested satellite cells have high Notch activity (purple line), which maintains Pax7 and inhibits *Myod* (indirectly: dotted line) and *Myogenin* (directly via Hey1) expression. Immediately after activation, satellite cells downregulate Notch activity and express *Myod* that is required for appropriate *Cdc6* expression and S-phase entry. During the amplification phase high Notch activity is restricted to upstream, Pax7^Hi^ cells that remain undifferentiated and self-renew to replenish the satellite cell pool. Notch activation is triggered by Dll1-bearing differentiating myoblasts. Non-muscle cells, like infiltrating inflammatory cells and fibro/adipogenic progenitors could also trigger or influence Notch activation. The expression of Dll ligands by the mature myofibres is likely, but remains to be demonstrated. *Lower panel: Rbpj* null satellite cells (no Notch activity: dotted purple line) enter the cell cycle normally and start proliferating. Mutant satellite cells differentiate faster (yellow cells) and fail to self-renew.

Notably, analysis of activated myoblasts isolated from regenerating muscles showed that endogenous Notch signalling is dramatically reduced upon activation, relative to the non-activated satellite cells [13,37]. In both studies, activated satellite cells from hindlimb muscles injured either by Notexin venom [13] or BaCl_2_ salt [37] were analysed by quantitative RT-PCR. Regardless of the injury model, the expression of the established Notch/Rbpj targets *Hey1*, *Hey2*, *HeyL Hes5*, and *Hes1* was reduced by up to 80-90% compared to quiescent satellite cells. Of note, only one Hes family member, *Hes6*, was found to be upregulated. However, *Hes6* is not a direct Notch/Rbpj target, and it promotes myogenic differentiation [59]. Importantly, microarray data comparing quiescent with activated satellite cells (isolated by FACS) *in vivo*, confirmed that Notch signalling was dramatically reduced in proliferating myoblasts [60]. Consistently, analysis of GFP^+^ cells from injured transgenic*Tg:Pax7-nGFP* mice showed that the reduction in Notch activity takes place almost immediately (within 20 hours) after injury [13], that is, before the first cell division occurs *in vivo *[50]. Subsequently, Notch signals weakly in the proliferating myoblasts and it starts to increase again by day 4–5 post-injury, a time point that correlates with the appearance of differentiated, Myogenin^+^ cells in the population and a decline in overall proliferation of the myogenic cells. We propose that the upregulation of Notch during regeneration, reflects the Pax7^Hi^, self-renewing myogenic cells. Maximal levels of Notch activity are then restored by days 20–30 post-injury, corresponding to tissue homeostasis and re-establishment of self-renewed satellite cells. Therefore, the majority of myogenic cells exhibit little to no Notch activity immediately following satellite cell activation at a time when these cells exit quiescence to undergo exponential expansion (Figure 1).

It is clear also that during regeneration, activated myoblasts interact with other cell types, including infiltrating inflammatory cells [61,62] and fibro/adipogenic progenitors [63]. These interactions might be productive in nature, at least in part, via Notch signalling. On this point, manipulations of the Notch pathway by injection of inhibitory or activating reagents in regenerating muscle can potentially affect myogenic and non-myogenic cell populations thereby resulting in indirect unrelated phenotypes. Moreover, discrepancies between compounds, or mutations targeting the *Notch* or *Rbpj* genes, could expose putative non-canonical functions, both of the receptors and Rbpj.

**Figure 2:**
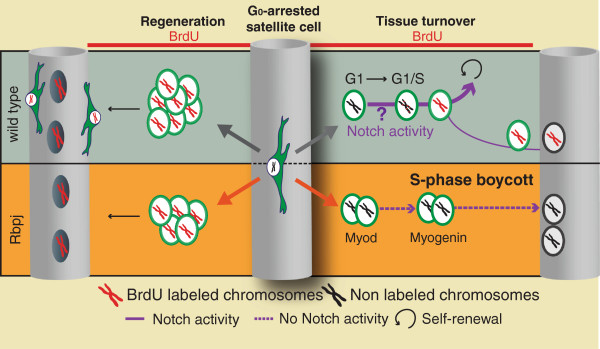
**Differential outcomes of loss of Notch activity during regeneration and tissue turnover.** During regeneration (left) both wild type and *Rbpj* null myoblasts proliferate and form new muscle fibres. In the absence of Notch signalling, the size of the amplified population is smaller since the cells fail to self-renew. During tissue homeostasis (right) a small fraction of satellites cells exit quiescence and divide (red chromosomes, BrdU^+^) to give rise to new satellite cells and fusion-competent myoblasts. Satellite cell specific deletion of *Rbpj* results in an increase in the number of cells that exit quiescence; these cells differentiate spontaneously. In sharp contrast to wild type cells the majority of *Rbpj* null satellite cells become Myogenin-positive without going through S-phase (black chromosomes).

In accordance with the expression data indicated above, genetic analysis of mice with activated or blocked Notch signalling support the view that elevated Notch signalling is not a requirement and likely it is incompatible with transit amplification of myoblasts. The dispensability of Notch activity during myoblast proliferation is illustrated by the phenotypes of *Rbpj* null mice in the context of regeneration. When muscles with *Rbpj* null satellite cells were injured (injury performed 16 days after tamoxifen-induced *Rbpj* gene deletion to ensure complete clearance of protein), tissue repair was overtly normal with only slightly smaller muscle fibres ([13], and data not shown; Figure 2). Moreover, the first mitosis of cultured *Rbpj* null satellite cells was similar in timing to control *Rbpj*+/- cells, as scored by videomicroscopy [13]. Furthermore, *Rbpj* null cells can amplify in culture for the first 3–4 days, that is, before significant differentiation takes place. In a complementary study, it was shown that quiescent satellite cells expressing constitutively activated Notch1 (*Pax7*^*CreERT2*^*: R26*^*stop-NICD*^), failed to exit from quiescence, as subsequent proliferation was significantly hindered [64]. Taken together, these data strongly suggest that a temporary decrease in Notch/Rbpj signalling at the time of satellite cell activation is a prerequisite for transit amplification of the myogenic population.

A seemingly contradictory observation is that freshly isolated canine satellite cells plated on Dll1 ligand, expanded more than non-treated cells [65]. One major difference between this and the *Pax7*^*CreERT2*^*: R26*^*stop-NICD*^ experiment cited above, is that cells were exposed to the ligand after their isolation, hence they were already activated. If Notch downregulation is indeed critical for the Myod-driven G1/S transition, it is possible that some of the dissociated cells were already engaged in the Myod program. Alternatively, chronic Notch signalling in culture might result in the eventual amplification of the cell population after an initial delay. These seemingly disparate observations might expose qualitative differences between the first G1/S transition, and those taking place during myogenic cell amplification.

## A model for Notch signalling during early satellite cell activation and homeostasis

In an attempt to rationalize the contextual roles of Notch signalling, we propose that the immediate collapse in activity following injury reflects the physical dissociation of satellite cells from the ligand source (likely the myofibre). The observation that pp38α/β and Myod are early markers of activated satellite cells is also coherent with a downregulation of Notch signalling, as both have been shown to be inhibited by activated Notch [35,51]. Indeed, it is reasonable to expect that Notch signalling should be transiently shut-off in order to allow the expression of Myod, whose activity via Cdc6 is important for exiting quiescence [53] (Figure 1). The increase in Notch activity at day 4–5 post-injury could be the outcome of signal emitting (Dll1^+^/Myog^+^) and signal receiving (Pax7^Hi^) cells, either implicating asymmetric or symmetric cell divisions. Analysis at the single-cell level would be required to investigate if the residual Notch activity scored in proliferating myogenic cells is restricted to a specific subpopulation, for example the self-renewing Pax7^Hi^ cells.

Notably, during homeostasis, loss of Rbpj activity results in depletion of the satellite cell pool after a delayed period of 5–7 weeks [13,37]. In stark contrast to activated satellite cells, the majority of the *Rbpj* null cells differentiate directly in the absence of muscle injury, without entering S-phase, then they fuse with the myofibres (Figure 2). This observation strongly suggests that in the context of normal satellite cell turnover, Notch activity is required for executing the G1/S transition. Analysis of Notch null clones in the Drosophila eye imaginal disc clearly demonstrates that Notch is required to overcome the G1-S check-point [66], consistent with the observations cited above. The finding that during regeneration *Rbpj* null satellite cells undergo, seemingly normally, the first G1/S phase following quiescence and they can transiently amplify, points to a secondary pathway that intervenes during injury, but that is absent during homeostasis. Once again, these contrasting behaviours underscore the distinct contextual outcomes of Notch activity in homeostasis vs. injury. Interestingly, in a model where constitutive expression of NICD was maintained in the upstream myogenic population (*Myf5*^*Cre*^-NICD), proliferating myogenic cells proceeded during development to express embryonic and foetal specific markers, then they prematurely exited the cell cycle to adopt a quiescent state late during foetal development. Here too, there are seemingly opposing behaviours for NICD: compatible with proliferating myogenic cells during early embryogenesis, and with the quiescent state in the late foetal phase [35]. These findings lead us to propose that Notch plays a role as facilitator, rather than an instigator, of cell fate decisions in this scenario, leaving the door open to explore alternative pathways that could promote stem cell quiescence.

## Towards understanding what lies downstream of Notch signalling

An important initial step towards understanding the developmental outputs of Notch signalling activation is to identify its downstream targets and the degree of conservation under diverse cellular contexts. In an attempt to address this question, a whole-genome ChIP-seq screen was performed for direct Rbpj/NICD targets in cultured myogenic cells, under activated or inhibited Notch signalling [11]**.** From this study, two points are pertinent to consider. First, in mouse muscle cells, binding of Rbpj to DNA is regulated dynamically by Notch signalling, thereby extending across-species, and at genome-wide scale, the binding behaviour of *Drosophila* Rbpj to the *E(spl)* gene cluster [12]. In other words, Rbpj is poorly bound, or not at all, to DNA in the absence of Notch signalling. By contrast, activation of Notch signalling results in recruitment of Rbpj together with NICD to enhancers/promoters, and subsequent activation of transcription. The dynamic association of Rbpj to DNA contradicted the prevalent view that this transcription factor statically occupies its binding sites, while exchanging repressors for activators in response to NICD. Unexpectedly, a second category of binding-sites was identified and these sequences were not co-occupied by NICD, but Rbpj was bound constitutively. Interestingly, this screen uncovered a number of Rbpj/NICD enhancers linked to genes encoding extracellular matrix associated proteins. This raises the intriguing possibility that skeletal muscle stem cells can partially contribute to the composition of their niche via cell-autonomous, Notch-regulated mechanisms. Such a mechanism is in agreement with a recent report that Notch signals control the homing of satellite cells by influencing the proper assembly of components in the basal lamina [67]. Moreover, myoblasts expressing constitutive activated Notch1 (*Myf5*^*Cre*^*:R26*^*stop-NICD*^), survive throughout development in the absence of myofibres, and in this scenario, cells leave the cell cycle in late foetal stages and are surrounded by laminin, possibly mimicking the niche [35].

## Review and conclusion

As often reported in the past, the outcome of Notch signals is profoundly dependent on the cellular context [68,69]. Muscle stem cells are no exception; pathway activity is critical for maintaining proliferating stem cells during growth, but also adult satellite cells, which are G_0_-arrested. Generally, Notch action is pro-myogenic when it is linked to inhibition of differentiation, but it can also suppress growth in tissues where its physiological role is to induce cell differentiation, for example in the skin [70,71]. The quiescent state, however, constitutes an alternative, more complex paradigm, where block of differentiation and reversible cell cycle arrest simultaneously co-exist. Once again, distinct extrinsic and intrinsic factors seem to determine the phenotypic consequences of loss of *Rbpj* function in satellite and adult neural stem cells, where the former differentiate without entering S-phase during homeostasis [13] and the latter collectively enter the cell cycle and undergo transit amplification [14,15]. A major challenge in the future is to identify the partners and the tissue specific downstream targets of Notch signalling that influence the developmental outcome of this fundamental signalling pathway, and to elucidate the unique and common outcomes of signalling through distinct Notch receptors in the context of Rbpj function.

## Competing interests

The authors declare that they have no competing interests.

## Authors’ contributions

PM and ST conceived the study and drafted the manuscript. Both authors read and approved the final manuscript.
